# Urinary Proteomics Analysis of Active Vitiligo Patients: Biomarkers for Steroid Treatment Efficacy Prediction and Monitoring

**DOI:** 10.3389/fmolb.2022.761562

**Published:** 2022-02-17

**Authors:** Yue-Tong Qian, Xiao-Yan Liu, Hai-Dan Sun, Ji-Yu Xu, Jia-Meng Sun, Wei Liu, Tian Chen, Jia-Wei Liu, Yan Tan, Wei Sun, Dong-Lai Ma

**Affiliations:** ^1^ Department of Dermatology, State Key Laboratory of Complex Severe and Rare Diseases, Peking Union Medical College Hospital, Chinese Academy of Medical Science and Peking Union Medical College, National Clinical Research Center for Dermatologic and Immunologic Diseases, Beijing, China; ^2^ Institute of Basic Medical Sciences, Chinese Academy of Medical Sciences, School of Basic Medicine, Peking Union Medical College, Beijing, China

**Keywords:** proteomic analysis, urine, active vitiligo, biomarkers, glucocorticoids resistance

## Abstract

Vitiligo is a common acquired skin disorder caused by immune-mediated destruction of epidermal melanocytes. Systemic glucocorticoids (GCs) have been used to prevent the progression of active vitiligo, with 8.2–56.2% of patients insensitive to this therapy. Currently, there is a lack of biomarkers that can accurately predict and evaluate treatment responses. The goal of this study was to identify candidate urinary protein biomarkers to predict the efficacy of GCs treatment in active vitiligo patients and monitor the disease. Fifty-eight non-segmental vitiligo patients were enrolled, and 116 urine samples were collected before and after GCs treatment. Patients were classified into a treatment-effective group (*n* = 42) and a treatment-resistant group (*n* = 16). Each group was divided equally into age- and sex-matched experimental and validation groups, and proteomic analyses were performed. Differentially expressed proteins were identified, and Ingenuity Pathway Analysis was conducted for the functional annotation of these proteins. Receiver operating characteristic curves were used to evaluate the diagnostic value. A total of 245 and 341 differentially expressed proteins between the treatment-resistant and treatment-effective groups were found before and after GCs treatment, respectively. Bioinformatic analysis revealed that the urinary proteome reflected the efficacy of GCs in active vitiligo patients. Eighty and fifty-four candidate biomarkers for treatment response prediction and treatment response evaluation were validated, respectively. By ELISA analysis, retinol binding protein-1 and torsin 1A interacting protein 1 were validated to have the potential to predict the efficacy of GCs with AUC value of 1 and 0.875, respectively. Retinol binding protein-1, torsin 1A interacting protein 1 and protein disulfide-isomerase A4 were validated to have the potential to reflect positive treatment effect to GCs treatment in active vitiligo with AUC value of 0.861, 1 and 0.868, respectively. This report is the first to identify urine biomarkers for GCs treatment efficacy prediction in vitiligo patients. These findings might contribute to the application of GCs in treating active vitiligo patients.

## Introduction

Vitiligo is a common acquired skin disorder characterized by solitary or multiple well-defined non-scaly depigmented skin patches, affecting 0.5%–2% of the world’s population (Taïeb and Picardo, 2009; Vitiligo Working Group, 2017). The clinical course of vitiligo is unpredictable, and patients experience alternating periods of stability and rapid disease progression (Dodiuk-Gad et al., 2015). It has been proposed that a combination of biochemical, environmental and immunological factors in genetically predisposed patients may contribute to the pathophysiology of vitiligo (Boniface et al., 2018). The administration of systemic immunosuppressants is considered an effective treatment option for active vitiligo (Boniface et al., 2018). Glucocorticoids (GCs) reduce T lymphocyte activity, suppress B cell antibody responses and inhibit the production of diverse cytokines in vitiligo patients (Dodiuk-Gad et al., 2015). Therefore, GCs are widely used to inhibit the rapidly progressive stage of vitiligo and stimulate repigmentation (Vitiligo Working Group, 2017). However, 8.2–56.2% of patients failed to achieve complete inhibition of lesions in previous studies (Pasricha and Khaitan, 1993; Radakovic-Fijan et al., 2001; Kanwar et al., 2013; Liu et al., 2020), which not only limits the therapeutic effect but also lead to hypothalamic–pituitary–adrenal axis suppression, osteoporosis, osteonecrosis, growth retardation, metabolic abnormalities and infections, resulting in serious consequences (Jackson et al., 2007). Moreover, there is a lack of effective biomarkers that can accurately evaluate treatment response or disease status. Finding a reliable and non-invasive marker is helpful for physicians to predict the sensitivity of systemic steroids therapy, develop personalized treatment plans and avoid severe drug-related adverse effects.

Mass spectrometry-based proteomic analysis is a powerful biological approach for the large-scale screening of disease-related protein biomarkers (Rodríguez-Suárez et al., 2014). Proteomics has been used to identify biomarkers for different skin diseases, such as psoriasis (Chularojanamontri et al., 2019), lupus erythematosus, systemic sclerosis (Trcka and Kunz, 2006), melanoma (Shields et al., 2016), graft-versus-host disease (Presland, 2017) and cutaneous T-cell lymphoma (Ion et al., 2016). Different biospecimens had been used to predict treatment efficacy in many diseases (Maher et al., 2011; Lu et al., 2012). In postherpetic neuralgia cerebrospinal fluid, the differential proteins before and after intrathecal methylprednisolone treatment were identified by 2D-DIGE analysis, and lipocalin-type prostaglandin D synthase was found to be down-regulated after effective treatment (Lu et al., 2012). In esophageal cancer, serum levels of C4a and C3a were found as predictive biomarkers of neoadjuvant chemoradiotherapy with a sensitivity and specificity of 78.6% and 83.3% by using Surface-enhanced laser desorption/ionization time-of-flight mass spectrometry (Maher et al., 2011). Among all kinds of body fluids proteome, urinary proteome research has the advantages of simplicity, noninvasive, rapid and large sample volume, and can be used for routine monitoring of patients (Shao et al., 2011). According to previous reports, urinary proteome had been used as early diagnostic markers of various diseases, including gliomas, type 2 diabetic nephropathy, pediatric medulloblastoma, etc (Guo et al., 2015; Hao et al., 2020; Wu et al., 2020). Urinary proteomics had also been used to predict the efficacy of drug therapy. Urinary vitamin D-binding proteins detected by two-dimensional gel electrophoresis could be a useful biomarker for predicting irbesartan treatment response in IgA nephropathy patients with good accuracy of 75% (Zeng et al., 2016). A set of proteins identified by 2-D PAGE and confirmed by western blot could distinguish IgA nephropathy patients responsive to angiotensin converting enzyme inhibitors from those unresponsive to the inhibition of renin-angiotensin system (Rocchetti, et al., 2008).

To date, limited studies have focused on the changes in proteomics profiles of vitiligo patients. Li Y. L. et al. (2018) reported that peroxiredoxin-6, apolipoprotein L1, apolipoprotein E and mannose-binding protein were differentially expressed between stable and progressive stages in vitiligo patients using two-dimensional gel electrophoresis coupled with mass spectrometry. Another proteomics studies revealed that several autoimmunity, lipid metabolism, oxidative stress, ion-dependent, and serine-type inhibitor proteins might be involved in the pathogenesis of vitiligo (Li Y.-L. et al., 2018). Liang et al. (2019) found elevated levels of CXCL4 and CXCL7 in the sera of vitiligo patients by high-performance liquid chromatography and tandem mass spectrometry analyses. Previous metabolic study found that the levels of catecholamine and 5-hydroxyindoleacetic acid in plasma and urine of patients with vitiligo (*n* = 20) were higher than those of normal controls (*n* = 20), suggested these monoamines may be the initiating event in the pathogenesis of vitiligo (Shahin et al., 2012). In our previous study, we found that there were significant differences in urinary metabolites between normal people and active vitiligo. By untargeted urinary metabolomic analysis, 71 differential metabolites were found and they enriched multiple pathways related to the pathogenesis of vitiligo (Liu et al., 2020). Above studies suggested that urine proteome and metabolome might be used to find the steroid treatment biomarkers of active vitiligo.

In the present study, we aimed to identify urinary biomarkers that could predict and monitor the efficacy of steroid treatment. Urine samples were collected from the treatment-resistance group and treatment-effective group before and after GCs treatment and randomly divided into a discovery group and validation group. Differentially expressed proteins (DEPs) were determined using proteomic analysis and further analyzed by Ingenuity Pathway Analysis (IPA) in the discovery group. In the validation group, biomarkers that could predict and monitor the effect of GCs therapy were identified, and receiver operating characteristic (ROC) curves were used to evaluate their diagnostic value. The differential proteins were further validated by enzyme-linked immunosorbent assay (ELISA). We believe that these results have profound significance for the determination of GCs treatment responses in patients with active vitiligo.

## Materials and Methods

### Patients

In this study, active non-segmental vitiligo patients were recruited from the Peking Union Medical College Hospital (PUMCH). Informed consent was obtained from all the participants and also from the legal guardians of participants less than 18 years of age. The study was approved by the local ethical committee of PUMCH, China (No. JS-2146). The diagnosis of vitiligo and assessment of disease activity was made by two experienced dermatologists based on the typical clinical presentation of depigmented lesions and wood lamp images. Patients with the emergence of new lesions, the expansion of original lesions or the occurrence of Koebner phenomenon within 6 months according to the Vitiligo Disease Activity score (Kohli et al., 2015) were enrolled. Exclusion criteria for the above patients were as follows: use of glucocorticoids or immunosuppressive agents in the last 3 months, contraindications for systemic prednisone, severe infection and malignant tumors.

All enrolled patients were given prednisone tablets at 0.5 mg/kg/day orally and the dose was tapered gradually within 5 weeks. The patients were asked to undertake a follow-up visit in the fifth week. A total of 20 ml of midstream urine samples on an empty stomach were collected at the time of diagnosis (before GCs treatment) and follow-up visit (after GCs treatment) from all subjects. Their treatment response was assessed and recorded by digital follow-up photographs, wood lamp images, and clinical examination results obtained at the last visit. The treatment-effective group was defined as all the pre-existing lesions achieve complete arrest and no appearance of new lesions, with or without re-pigmentation. The treatment-resistant group was defined as the lesions continued to spread.

A total of 58 patients were enrolled and they were randomly divided into two groups: the discovery group (29 samples: 8 from treatment-resistant group, 21 from treatment-effective group) and the validation group (29 samples: 8 from treatment-resistant group, 21 from treatment-effective group). In total, 116 urinary samples were collected. These samples were further evaluated by routine urine tests to exclude related diseases. Once collected, the urine samples were stored at −80°C as soon as possible.

### Sample Preparation

Each sample was taken 100 μL to pool into a pooled sample. The pooled sample was used as quality control (QC). QC sample was injected frequently to monitoring reproducibility of the LC-MS/MS. For each group, a pooled sample of equal amounts of urine from each sample was used for library generation. The urine samples and pooled samples were precipitated overnight using three times the volume of ethanol at 4°C. Then, the pellets were centrifuged at 10,000 × g for 30 min and resuspended in lysis buffer (7 M urea, 2 M thiourea, 0.1 M of DTT, and 5 mM of Tris, pH = 8). The protein concentration of the urine samples was determined by the Bradford method. Protein digestion was carried out using the filter-aided sample preparation technique (FASP) method. The proteins were denatured by incubation with 20 mM dithiothreitol at 95°C for 5 min and then were alkylated in 55 mM iodoacetamide in the dark for 45 min. Trypsin (1:50) was added to these samples and then were incubated at 37°C overnight. After digestion, the peptides were desalted with a C18 solid-phase extraction column (Waters Oasis, Ireland), washed with 500 μL of 0.1% formic acid and eluted with 500 μL of 100% ACN and then vacuum-dried.

### High-pH Reversed-Phase Liquid Chromatography Separation

The pooled peptide was separated by high-pH RPLC columns (4.6 mm × 250 mm, C18, 3 μm; Waters, USA). The pooled sample was loaded onto the column in buffer A1 (H_2_O, pH 10). The elution gradient was 5%–30% buffer B1 (90% ACN, pH 10; flow rate, 1 ml/min) for 30 min. The eluted peptides were collected at one fraction per minute. Then, the thirty fractions were vacuum-dried. The 30 fractions were re-suspended in 0.1% formic acid and then were concatenated into 10 fractions by combining fractions 1, 11, 21, and so on.

### Liquid Chromatography-Tandem Mass Spectrometry Analysis

The Orbitrap Fusion Lumos Tribrid (Thermo Scientific, Bremen, Germany) coupled with an EASY-nLC 1000 was used for LC–MS/MS analysis. The digested peptides were dissolved in 0.1% formic acid and separated on an RP C18 self-packing capillary LC column (75 μm × 150 mm, 3 μm). The eluted gradient was 5%–30% buffer B2 (0.1% formic acid, 99.9% ACN; flow rate, 0.3 μL/min) for 60 min.

To generate the spectral library, the fractions from RPLC were analyzed in the data-dependent acquisition (DDA) mode. The parameters were set as follows: the MS was recorded at 350–1,500 m/z at a resolution of 60,000 m/z; the maximum injection time was 50 ms, the auto gain control (AGC) was 1e6, and the cycle time was 3 s. MS/MS scans were performed at a resolution of 15,000 with an isolation window of 1.6 Da and a collision energy at 32% (HCD); the AGC target was 50,000, and the maximum injection time was 30 ms.

Each sample and the QC samples were analyzed in the data-independent acquisition (DIA) mode. For MS acquisition, the variable isolation window DIA method with 38 windows was developed. The specific window lists were constructed based on the DDA experiment of the pooled sample. The precursor ion number was equalized in each isolation window based on the precursor m/z distribution of the pooled sample. The full scan was set at a resolution of 120,000 over the m/z range of 400–900, followed by DIA scans with a resolution of 30,000; the HCD collision energy was 32%, the AGC target was 1E6, and the maximal injection time was 50 ms.

### Spectral Library Generation

To generate a comprehensive spectral library, the pooled sample from each group was processed. The DDA data were processed using Proteome Discoverer 2.3 (Thermo Scientific, Germany) software and searched against the human Swiss-Prot database (*Homo sapiens*, 20205 SwissProt, 2019-05 version) appended with the iRT fusion protein sequence (Biognosys). A maximum of two missed cleavages for trypsin was used, cysteine carbamidomethylation was set as a fixed modification, and methionine oxidation (+ 15.995 Da), asparagine and glutamine deamidation (+ 0.984 Da), lysine carbamylation (+ 43.006 Da) were used as variable modifications. The parent and fragment ion mass tolerances were set to 10 ppm and 0.02 Da, respectively. The applied false discovery rate (FDR) cutoff was less than 1% at the protein level. The results were then imported to Spectronaut Pulsar (Biognosys, Switzerland) software to generate the library. Spectronaut Pulsar software allows the generation, merging, and management of spectral libraries. Next, we merged the four libraries to one urine spectral library, which contained all the information generated from the different libraries.

### Data Analysis

The DIA raw data were loaded to the Spectronaut Pulsar software to calculate peptide retention time based on iRT data. And Spectronaut provided protein identification and quantitation by matching the retention time, m/z etc to peptide library. The retention time prediction type was set to dynamic iRT, and interference correction at the MS2 level was enabled. The MS1 and MS2 tolerance strategy was set to dynamic. It applied a correction factor to the automatically determined mass tolerance. The correction factor for ms1 and ms2 was all set as 1. The precursor posterior error probability (PEP) cut-off was set to 1. And precursors that do not satisfy the cut-off will be imputed. The top N (min: 1; max: 3) precursors per peptide was used for quantify calculation. The top N ranking order is determined by a cross-run quality measure. Peptide intensity was calculated by summing the peak areas of their respective fragment ions for MS2. Cross-run normalization was enabled to correct for systematic variance in the LC-MS performance, and a local normalization strategy was used. Normalization was based on the assumption that on average, a similar number of peptides are upregulated and downregulated, and the majority of the peptides within the sample are not regulated across runs and along retention times (Callister et al., 2006). Protein inference, which gave rise to the protein groups, was performed on the principle of parsimony using the ID picker algorithm as implemented in Spectronaut Pulsar. All results were filtered by a Q value cutoff of 0.01 (corresponding to an FDR of 1%). Protein intensity was calculated by summing the intensity of their respective peptides. Proteins identified in more than 50% of the samples in each group were retained for further analysis. Missing values were imputed based on the k-nearest neighbor method. Pattern recognition analysis [principal component analysis (PCA) and orthogonal partial least squares analysis (OPLS-DA)] was carried out using SIMCA 14.0 software (Umetrics, Sweden) to visualize group classification. A total of 50 permutation tests were used to validate the OPLS-DA model to avoid over-fitting of the model. Non-parameter Wilcoxon rank-sum test was performed for significance evaluation of proteins between groups. The proteins that presented a *p*-value <0.05 were considered DEPs.

### Protein Function Annotation

All the differential proteins were used for pathway analysis using Ingenuity Pathway Analysis software (Ingenuity Systems, Mountain View, CA) for functional analysis. The Swissport accession numbers were uploaded to IPA software (QIAGEN). The proteins were mapped to disease and function categories and canonical pathways available in Ingenuity and other databases and were ranked by *p*-values.

### ELISA Assay

Five selected candidate biomarkers, namely retinol binding protein (RBP)-1, torsin 1A interacting protein (TOR1AIP)-1 and protein disulfide-isomerase A (PDIA)-4, s-adenosylmethionine synthase isoform type-2 and envoplakin were assayed with commercially available enzyme-linked immunosorbent assays according to the manufacturer’s instructions (Quanzhou jiubang Biotechnology Co., Ltd.) in the treatment-resistant group (*n* = 10) and the treatment-effective group (*n* = 12) before and after GCs treatment and health controls (total *n* = 12) using ELISA. Optical densities at 450 nm were measured using a microplate reader (SpectraMax cmax plus, Molecular Devices).

## Results

**FIGURE 1:**
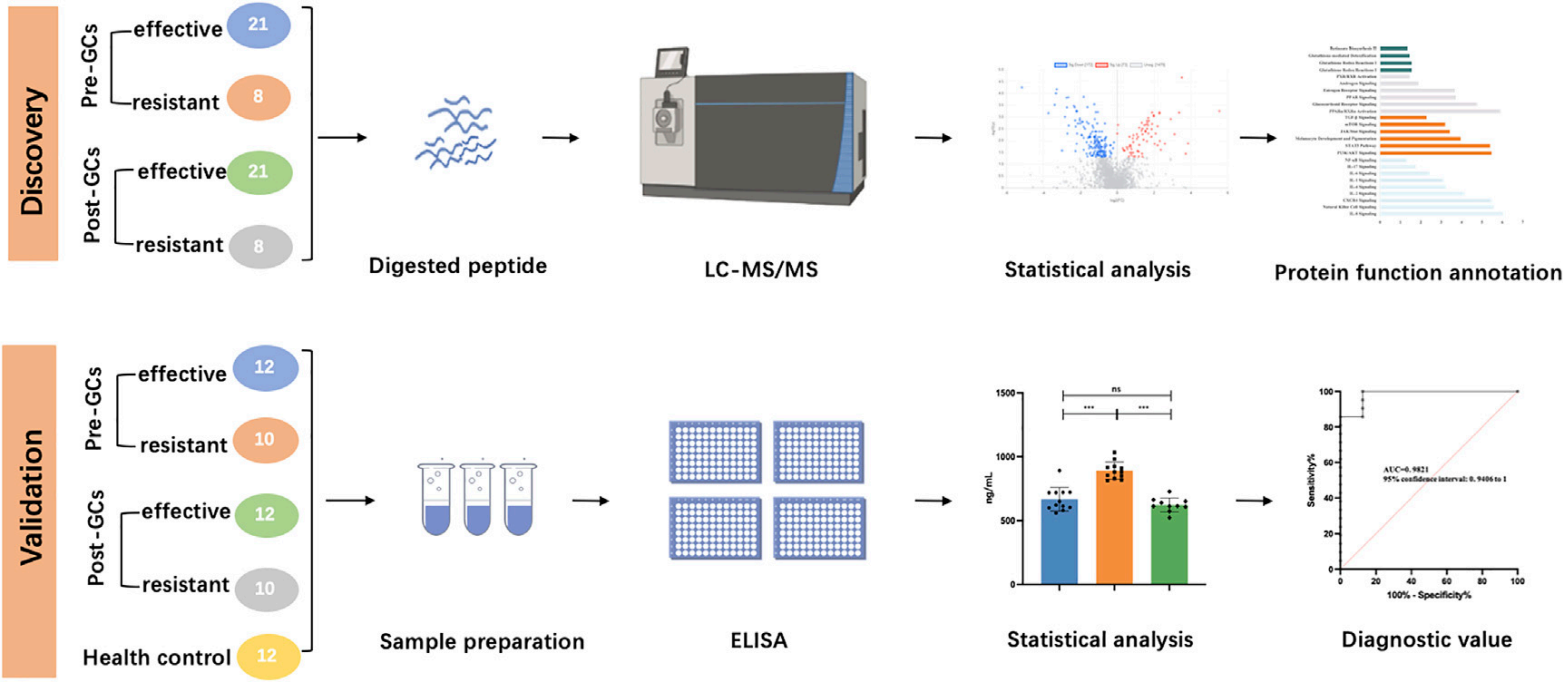
Workflow of this study.

In this study, 58 advanced non-segmental vitiligo patients were recruited, including 16 treatment-resistant patients and 42 treatment-effective patients. The clinical characteristics of all participants are shown in Table 1 (detailed information is provided in Supplementary Table S1). The age, sex, disease severity, disease duration and other indexes showed no significant differences between the two groups. No related diseases were detected in routine urine tests. All enrolled patients were classified into a treatment-resistant group (*n* = 16) and a treatment-effective group (*n* = 42). Each group was divided equally into an age- and sex-matched experimental group for differential protein identification and a validation group to verify biomarkers for predicting GCs treatment responses and monitoring treatment effects. Urinary proteomic analysis of the treatment-resistant group and treatment-effective group before and after treatment was performed. The differential proteins were further analyzed in the validation groups. To validate the results from DIA analysis, 10 treatment-resistant group, 12 treatment-effective group before and after GCs treatment and 12 health controls were included for ELISA analysis (Detailed information in Supplementary Table S1). The workflow of this study is shown in Figure 1.

**TABLE 1:** Demographics of active vitiligo patients enrolled in this study.

	Discovery cohort (*n* = 29)			Validation cohort (*n* = 29)		
	Effective (*n* = 21)	Resistant (*n* = 8)	P^*^	Effective (*n* = 21)	Resistant (*n* = 8)	P^#^
Average age (years)	23.62 ± 12.97	22.00 ± 15.49	0.79	23.52 ± 14.62	20.75 ± 12.94	0.65
Disease duration (years)	4.66 ± 4.47	3.25 ± 1.56	0.41	4.28 ± 4.71	4.56 ± 6.72	0.90
Sex						
Female	9	4	—	9	3	—
Male	12	4		12	5	
Disease subtype						
Non-segmental	21	8	—	21	8	—
Disease severity						
1%BSA	66	5	—	11	4	—
1–5%BSA	11	3		6	4	
6–50%BSA	4	0		4	0	
Family history						
	3	0	—	3	1	—
Comorbidity						
	3	0	—	0	0	—

BSA: body surface area; *p-value of test comparing effective group with resistant group in discovery cohort; #*p*-value of *t*-test comparing effective group with resistant group in validation cohort.

### Database: A Comprehensive Profile of the Proteome

A comparative proteomic analysis of all 116 urine samples was performed to investigate the differences between each group. Samples were analyzed randomly. A QC standard was prepared as a pooled mixture of aliquots from urine samples representative of each group. The QC sample was injected five times before and frequently throughout the analytical run to monitor instrument stability. Overall, 10 injections were performed during the entire analysis. The QC samples showed a stable condition with a high correlation (*R* ^2^ = 0.95) (Supplementary Figure S1). In total, 2885 proteins were identified. The identification of 2199 proteins was accepted at a FDR <1.0% by analyzing the levels of proteins and peptides formed by ≥2 unique peptides (Supplementary Table S2).

### Identification of Biomarkers for Predicting GCs Treatment Responses

**FIGURE 2:**
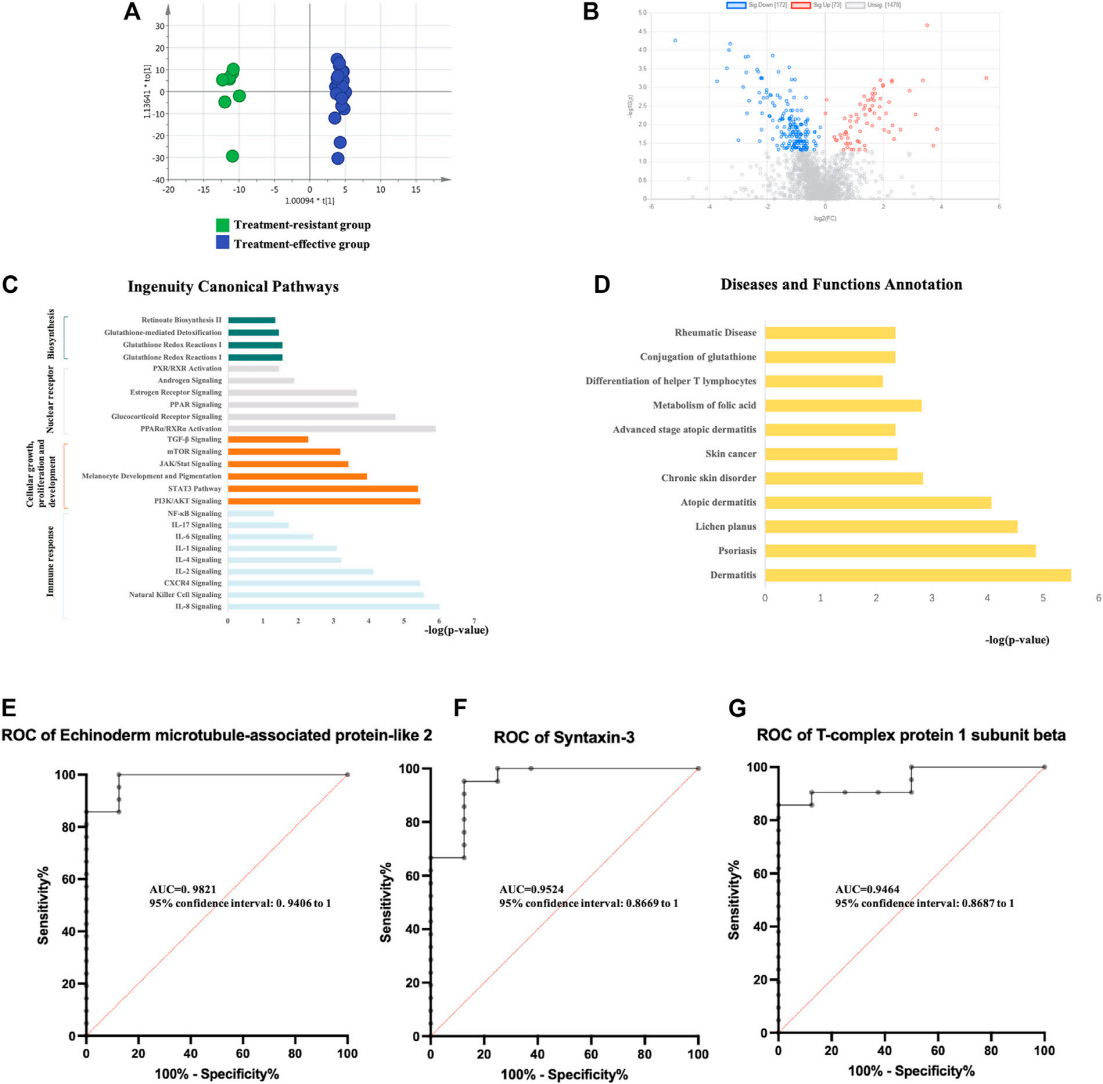
Analysis of urine proteomics between treatment-resistant and treatment-effective active vitiligo patients before GCs. **(A)** Score plot of OPLS-DA model showed good separation. (SIMCA 14.0 software, Umetrics, Sweden) **(B)** Volcano plot of differential expressed proteins. (Non-parametric tests) **(C)** Ingenuity canonical pathway. **(D)** Diseases and functions. The ROCs of Echinoderm microtubule-associated protein-like 2 **(E)**, Syntaxin-3 **(F)** and T-complex protein 1 subunit beta **(G)** were analyzed between baseline of treatment resistant/effective groups.

Twenty-nine urinary samples collected at baseline (8 treatment-resistant and 21 treatment-effective patients) were used for the proteomic analysis of treatment effects. First, to explore the proteomic profiling differences between these groups, unsupervised PCA was performed. The score plot showed that the treatment-resistant group was separated from the treatment-effective group (Supplementary Figure S3). Second, OPLS-DA was performed to further determine the proteomic differences between these groups. The treatment-resistant group and treatment-effective group were separated from each other in the OPLS-DA model (Figure 2A).

Differential proteins were identified based on adjusted *p*-values <0.05. Overall, 245 DEPs were identified, with 73 proteins upregulated and 172 proteins downregulated in the treatment-resistant group compared with the levels in the treatment-effective group (Figure 2B, Supplementary Table S3). The DEPs were further analyzed by IPA. In the disease and biofunction analysis, the DEPs were enriched in folic acid metabolism, immune responses, dermatological diseases (dermatitis, psoriasis, lichen planus, and atopic dermatitis) and connective tissue diseases (Figure 2C, Supplementary Table S3). Canonical pathway analysis revealed that these proteins were involved in nuclear receptor signaling, cellular immune response and cytokine signaling, cellular growth, proliferation and development growth factor signaling and metabolism-related pathways (Figure 2D, Supplementary Table S3).

To discover potential biomarkers for predicting the efficacy of GCs treatment, the proteomic analysis of the validation group (8 treatment-resistant patients and 21 treatment-effective patients) was performed. Eighty DEPs were verified in the validation group. The diagnostic accuracy of the identified DEPs between the two groups with different treatment efficacies was further evaluated (Supplementary Table S3). In the validation group, all 80 proteins showed potential diagnostic value with an area under the curve (AUC) above 0.7, 42 proteins had diagnostic value with an AUC above 0.8, and 9 proteins exhibited good diagnostic value with an AUC above 0.9. Echinoderm microtubule-associated protein-like 2, syntaxin-3 and T-complex protein 1 subunit beta showed the highest potential predictive ability with AUCs of 0.98214, 0.95238, and 0.94643, respectively (Figures 2E–G).

### Identification of Biomarkers for Monitoring GCs Treatment Responses

**FIGURE 3:**
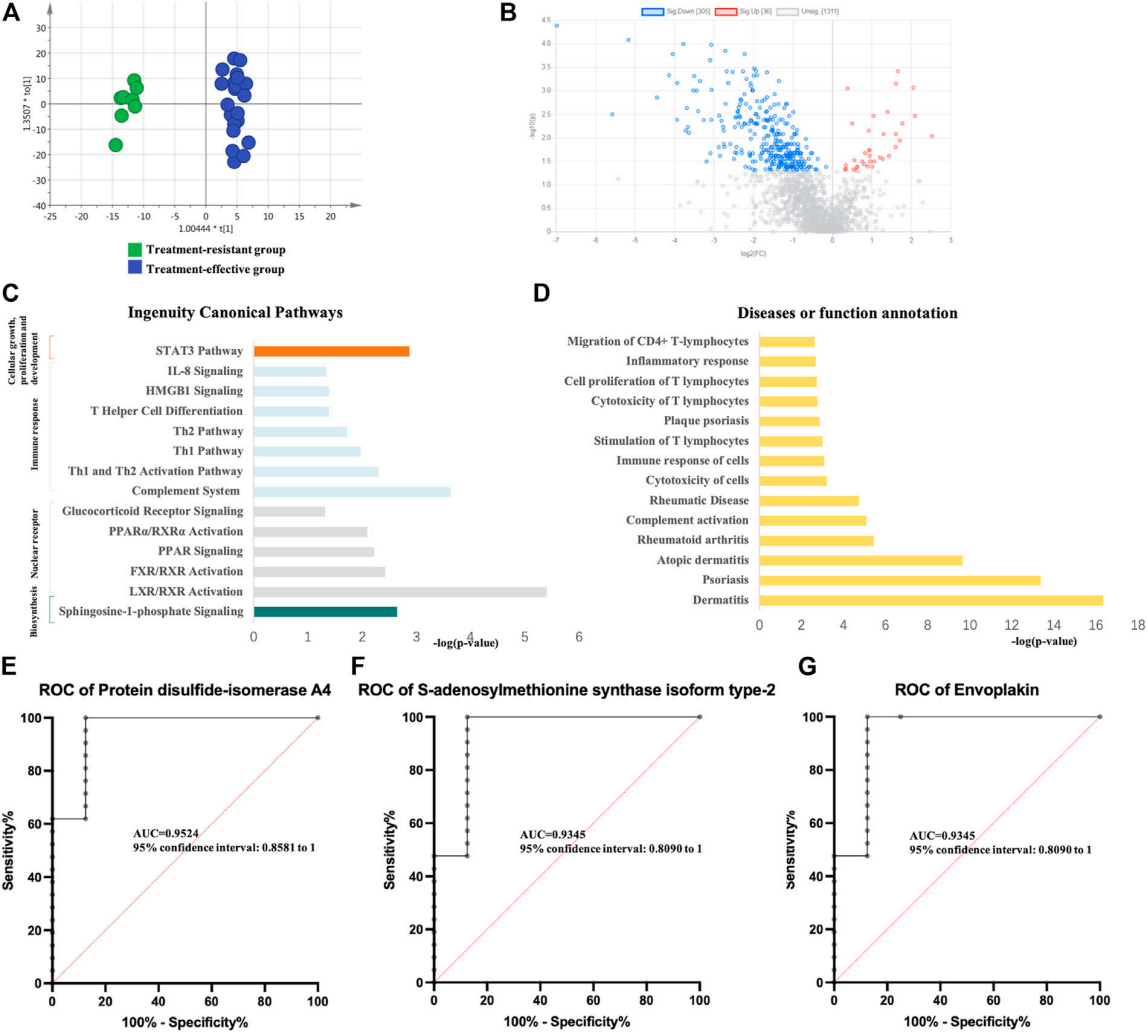
Analysis of urine proteomics between treatment-resistant and treatment-effective active vitiligo patients after GCs. **(A)** Score plot of OPLS-DA model showed good separation. (SIMCA 14.0 software, Umetrics, Sweden) **(B)** Volcano plot of differential expressed proteomics. (Non-parametric tests) **(C)** Ingenuity canonical pathway. **(D)** Diseases and functions. The ROCs of Protein disulfide-isomerase A4 **(E)**, S-adenosylmethionine synthase isoform type-2 **(F)** and Envoplakin **(G)** were analyzed between follow-up of treatment resistant/effective groups.

Using the same method as above, the expression of differential proteins between the two groups after GCs treatment was detected. PCA and OPLS-DA analysis (Figure 3A) showed significant differences between the two groups. Specifically, 341 DEPs were identified (36 upregulated and 305 downregulated) (Figure 3B, Supplementary Table S4). Pathway enrichment analysis showed significant enrichment of immune-related pathways, cellular growth, proliferation and development and intracellular and second messenger signaling (Figure 3C). In the disease and biofunction analysis, the DEPs were enriched in stimulation, cytotoxicity and cell proliferation of T lymphocytes, dermatological diseases (dermatitis, psoriasis, lichen planus, and atopic dermatitis) and connective tissue diseases (Figure 3D). In the validation group, 54 DEPs with a predictive value of 0.7 were identified. Thirty DEPs had good diagnostic value with an AUC above 0.8, and 6 DEPs exhibited good diagnostic value with an AUC above 0.9 (Supplementary Table S4). Protein disulfide-isomerase A4, S-adenosylmethionine synthase isoform type-2 and envoplakin showed the highest potential predictive ability with AUCs of 0.95238, 0.93452 and 0.93452, respectively (Figures 3E–G).

### ELISA Validations

RBP-1, TOR1AIP-1, and MAT2A were selected as candidate biomarkers for steroids efficacy prediction before treatment. PDIA-4, MAT2A, EVPL, and TOR1AIP-1 were selected as candidate biomarkers for treatment outcome evaluation after treatment. The ELISA results for above proteins were consistent to those in DIA analysis (Table 2, Supplementary Table S5, Supplementary Figure S4).

**TABLE 2:** Comparison of differential protein by enzyme-linked immunosorbent assay and DIA analysis.

Var ID (primary)	Gene	Name	DIA fold change	DIA AUC	ELISA fold change	ELISA AUC
Before treatment						
P09455	RBP1	retinol binding protein 1	0.28566	0.82738	0.6981	1
P31153	MAT2A	methionine adenosyltransferase 2A	0.14897	0.96429	0.8876	1
Q5JTV8	TOR1AIP1	torsin 1A interacting protein 1	3.2949	0.88095	1.2354	0.875
After treatment						
P13667	PDIA4	protein disulfide isomerase family A member 4	0.26298	0.84524	0.9156	0.9667
P31153	MAT2A	methionine adenosyltransferase 2A	0.21488	0.84524	0.9156	0.9
Q92817	EVPL	envoplakin	0.30491	0.86905	0.906	0.7917
Q5JTV8	TOR1AIP1	Torsin 1A interacting protein 1	0.35787	0.77381	0.7936	1

DIA, date-independent acquisition; PRM, parallel reaction monitoring; AUC, area under curve.

**FIGURE 4:**
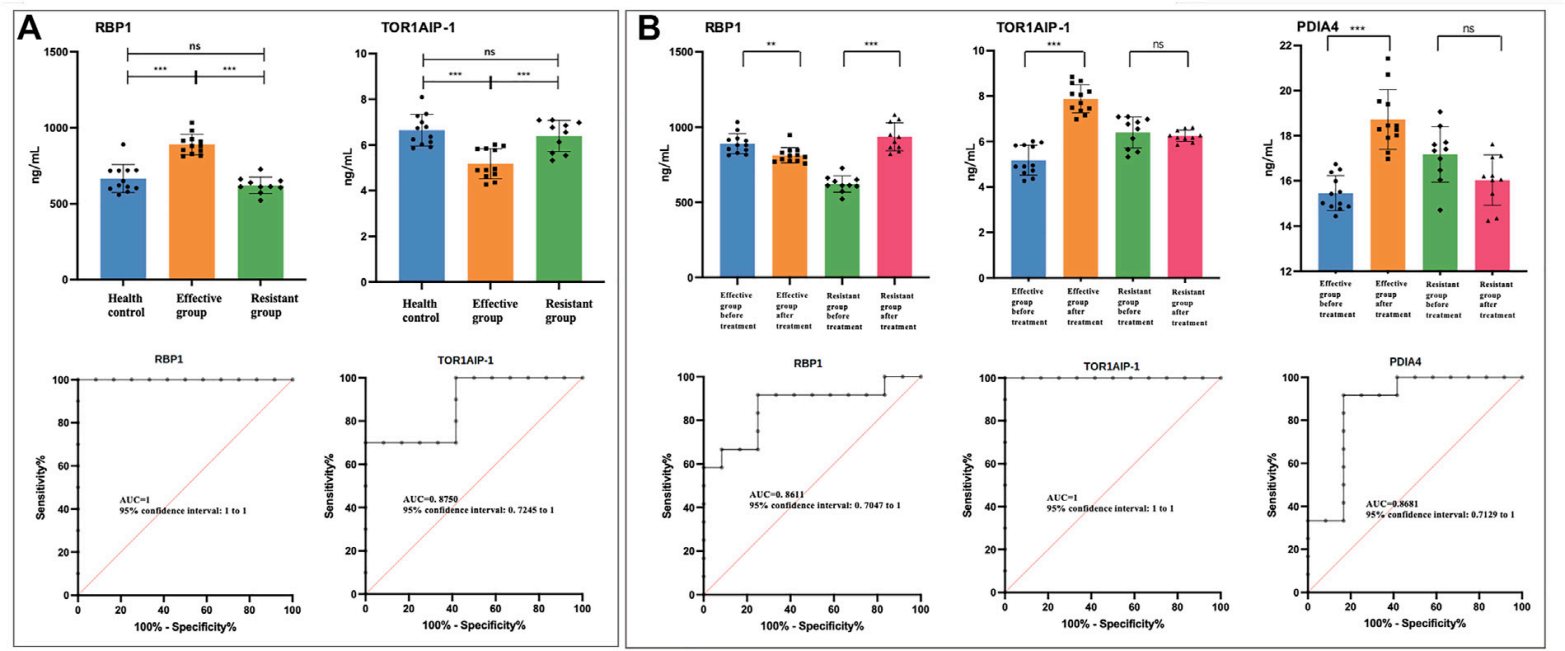
The urinary level of differential proteins measured by enzyme-linked immunosorbent assay. The asterisks indicate the level of significance. * *p* < 0.05; ** *p* < 0.01; *** *p* < 0.001; ns, no statistical difference. RBP-1, retinol binding protein-1; TOR1AIP-1, torsin 1A interacting protein-1; PDIA-4, protein disulfide-isomerase A-4; MAT2A, S-adenosylmethionine synthase isoform type-2; EVPL, evoplakin. **(A)** Before treatment, the comparison of RBP-1 and TOR1AIP-1 in treatment effective/resistant group and normal control. The ROCs were analyzed between treatment effective and resistant groups before treatment. **(B)** The comparison of RBP-1, TOR1AIP-1, and PDIA-4 in treatment effective/resistant group before and after treatment. The ROCs were analyzed in treatment-effective groups before and after treatment.

According to compare the results of treatment-effective/resistant group and normal control, PDIA-4, MAT2A and EVPL showed statistical significance (Supplementary Figure S5A). But it could not predict the treatment response according to compare the vitiligo patient (treatment-effective or -resistant) and health control. For RBP-1 and TOR1AIP-1, the treatment-resistant group and normal control showed no difference, but treatment-sensitive group and normal control showed statistical significance (Figure 4A). Therefore, it might be possible to predict the treatment response according to the RBP-1 and TOR1AIP-1 level in urine, which might be used as treatment prediction biomarker. The ROC curve was used to evaluate the predictive effect of differential proteins on glucocorticoids treatment resistance. The results showed that RBP-1 and TOR1AIP-1 had a predictive AUC value of 1 and 0.875 respectively (Figure 4A).

To validate the urinary biomarkers for evaluating treatment efficacy, the levels of five DEPs were compared between the treatment effective/resistant groups (Figure 4B, Supplementary Figure S5B). In treatment effective group, five proteins showed statistical difference after treatment, but in treatment resistant group, TOR1AIP-1 and PDIA4 showed no statistical difference (Figure 4B, Supplementary Figure S5B). Considering the trend change of five proteins in the treatment effective/resistant groups, RBP-1, TOR1AIP-1, and PDIA4 showed opposite trends (Figure 4B). These results suggested that RBP-1, TOR1AIP-1, and PDIA4 might be used to evaluate the GCs treatment. The ROC curve showed that RBP-1, TOR1AIP-1, and PDIA-4 had a predictive AUC value of 1 and 0.861, 1 and 0.868 respectively (Figure 4B).

## Discussion

GCs administration is an important therapeutic intervention in several inflammatory, autoimmune, allergic and lymphoproliferative diseases (Dodiuk-Gad et al., 2015). The activity of GCs is regulated by the glucocorticoid receptor (GR) (Hapgood et al., 2016). In particular, GR isoform *α* mediates the physiological and pharmacological actions of GCs. GR isoform *ß* functions as a dominant-negative inhibitor and antagonizes the activity of GRα. Resistance to GCs has been reported in patients with inflammatory bowel disease, interstitial lung diseases, asthma, immune thrombocytopenia and autoimmune diseases (Farrell and Kelleher, 2003). The mechanism of GCs resistance may be related to genetic susceptibility, cytokine activation of mitogen-activated protein kinase (MAPK) pathways, increased GRβ expression, excessive activation of activator protein-1 and increased p-glycoprotein-mediated drug efflux (Barnes and Adcock, 2009). The mechanism of resistance to GCs therapy in patients with vitiligo has not been investigated. Some biomarkers have been proposed for the prediction or monitoring of vitiligo treatment responses, but their discriminatory power has not been described. Hwang et al. (1999) reported that the serum level of soluble intercellular adhesion molecule-1 was significantly decreased after systemic steroid treatment in the clinically improved group. El-Domyati et al. (2021) revealed that the levels of C-X-C motif chemokine ligand (CXCL) 10 were decreased in the serum and lesions of vitiligo patients after systemic steroid treatments. In this study, we identified several DEPs that can be used to predict and evaluate the therapeutic effect in active vitiligo patients via urine proteomic analysis. These findings might contribute to the application of GCs in treating active vitiligo patients in the future.

### Biomarkers in Predicting GCs Efficacy in Active Vitiligo

Pathway enrichment analysis showed significant enrichment of several pathways related to the mechanisms of GCs resistance, including nuclear receptor signaling (GR signaling, peroxisome proliferator-activated receptor signaling, peroxisome proliferator-activated receptor alpha-retinoid X receptor alpha activation, pregnane X receptor-retinoid X receptor activation and androgen receptor signaling), cellular immune responses and cytokine signaling [interleukin (IL)-1/2/3/6/8/17 and nuclear factor-kappa B (NF-κB) signaling], cellular growth, proliferation and development growth factor signaling [transforming growth factor (TGF)-β, PI3K/AKT and Janus kinase/signal transduction and activator of transcription (JAK-STAT) signaling] and metabolism (vitamin A, sugar and glycine degradation). Some classic signaling pathways of vitiligo were also enriched, including melanocyte development and pigmentation signaling and the CXCR4 signaling pathway.

In our study, the NF-κB signaling pathway was enriched. NF-κB is expressed in a wide variety of cells and activated by pro-inflammatory cytokines, chemokines, stress-related factors and extracellular matrix degradation products (Rigoglou and Papavassiliou, 2013). Studies have found that NF-κB and GR mutually inhibit each other’s transcriptional activity through multiple mechanisms (Zappia and Monczor, 2019). For example, high levels of NF-κB attenuate GR function by blocking the GR signaling pathway, thereby affecting GCs responses (Zappia and Monczor, 2019). The negative effect of NF-κB on GR function may account for the development of GCs resistance in many diseases. It has been reported that increased expression of NF-κB decreased the responsiveness of cells to GCs in patients with corticosteroid refractory asthma (Adcock and Barnes, 2008). One study reported the *NF-κB* gene as a biomarker to predict GCs responsiveness in asthma with an accuracy of 81.25% (Hakonarson et al., 2005). Our study further indicates that NF-κB is related to GCs sensitivity.

In our study, the TGF-β signaling pathway was enriched. TGF-β and related growth factors are secreted pleiotropic factors that play critical roles in regulating cell proliferation, differentiation, death, migration and immune responses (Hata and Chen, 2016). A previous study reported that TGF-β induced fibroblast to myofibroblast transdifferentiation in a human lung fibroblast cell line and increased GRβ expression in myofibroblasts, which is responsible for GCs resistance in severe asthma (Breton et al., 2018). It has also been proposed that increased TGF-β signaling in blood cells might confer a good response to inhaled corticosteroids in patients with asthma (Kim et al., 2021). To date, studies on the relationship between TGF-β and GCs resistance have mainly focused on respiratory diseases, and the mechanisms contributing to GCs resistance in vitiligo require further research.

Some proteins previously reported to be related to GCs sensitivity and autoimmune diseases were identified in this study. For example, IL-2 receptor subunit alpha (IL-2Rα) was downregulated in the treatment-effective group before GCs treatment. IL-2Rα is the receptor for IL2, which is involved in the IL-2-mediated signaling pathway and other important immune responses and is related to GCs resistance (Kanagalingam et al., 2019). High levels of IL-2 have been found in the bronchoalveolar lavage fluid of steroid-resistant asthmatics (Leung et al., 1995). Previous reports revealed that IL-2 inhibited the transcriptional activity of GR *via* JAK-STAT and MAPK pathways in murine T lymphocytes and reduced GR responsiveness (Biola et al., 2001). Based on these facts, we propose that IL2Rα is related to the sensitivity of GCs treatment.

In our study, RBP-1 was downregulated in the treatment-effective group, which was consistent with previous studies. RBP is known to contribute to retinol uptake, storage and homeostasis *via* activation of the retinoic acid receptor (RAR) and retinol biosynthesis pathways (Farias et al., 2005). It has been reported that retinoic acid reduced GCs sensitivity and GR activities through a RAR-dependent mechanism in skeletal muscle cells (Aubry and Odermatt, 2009). Urinary RBP has been suggested as a stable biomarker to predict early proximal tubular dysfunction in nephrotic syndrome (Sesso et al., 1992). A previous study revealed that increased urinary levels of RBP were detected in the steroid-unresponsive group compared with the steroid-responsive group pre-steroid treatment in patients with idiopathic nephrotic syndrome by immunoenzymometric assays (Chehade et al., 2013). In addition, the RBP level predicted steroid treatment responsiveness with an AUC of 0.83 (Chehade et al., 2013). The exact roles of RBP in GCs resistance require further study. Our results showed that glutathione S-transferase theta (GSTT)-1 was upregulated in the treatment-effective group. Glutathione S-transferases (GSTs) are a multigene family of detoxification enzymes involved in cellular defenses against oxidative stress, chemical toxicities and therapeutic agents (Uhm et al., 2007). GSTs bind to GCs and are suggested to play a possible role in modulating the therapeutic effects of prednisone (Maung et al., 1994). For example, a positive correlation between GSH levels and prednisolone resistance has been reported. In addition, a case-control study revealed that acute lymphoblastic leukemia patients with a homozygous deletion of GSTT1 (null genotype) showed good prednisone responses (Anderer et al., 2000). Our study further confirmed that GSTT1 is related to effective GCs responses in the treatment of active vitiligo.

### Biomarkers in Evaluating GCs Efficacy in Active Vitiligo

To determine whether the urinary proteome reflected the pathological alternation in patients after GCs treatment, we used the DIA method to identify and verify DEPs. Pathway enrichment analysis of these DEPs showed significant enrichment in IL-2/3/4/8 signaling, CXCR4 signaling, Th1 and Th2 activation pathways, Th cell differentiation, T cell receptor signaling, B cell development, Th1 and Th2 pathways, STAT3 signaling, GC receptor signaling, α-adrenergic signaling and G protein signaling.

Several immune-related pathways associated with GCs resistance were enriched in our study. A previous study revealed that the level of the Th1-derived cytokines tumor necrosis factor (TNF)-α and interferon (IFN)-γ were increased in some severe asthma patients who were resistant to corticosteroid therapy (Britt et al., 2019). Exposure to TNF-α and IFN-γ reduces GR activity and GCs efficacy by altering GR phosphorylation and reducing interactions with transcriptional GR coactivators in airway cells (Bouazza et al., 2012). A previous study revealed that human lymphocytes and peripheral blood mononuclear cells treated with a combination of IL-2 and IL-4 showed impaired GRα nuclear translocation, binding affinity and GR transactivation function, resulting in reduced GCs responsiveness (Kam et al., 1993; Goleva et al., 2009). Therefore, GCs resistance may develop under the joint action of several inflammatory factors, cytokines and immune cell pathways.

STAT-3 pathways were enriched after GCs treatment in our study. GRs interfere with the STAT signaling pathway by interacting with STAT3 and STAT5 and affecting immune responses (Petta et al., 2016). It has been reported that escape from drug toxicity *via* STAT3 contributed to GCs resistance in lymphomas (Teng et al., 2012). The development of GCs resistance after long-term prednisolone treatment has been observed in human melanoma cells, which showed a two-fold increase in STAT3 expression (Krasil’nikov and Shatskaya, 2002). Therefore, we speculate that STAT3 pathway activation may be related to hormone therapy resistance.

Envoplakin is a component of desmosomes and the epidermal cornified envelope. Envoplakin is also a component of the antigen complex in paraneoplastic pemphigus (an autoimmune blister skin disease). It has been reported that the level of envoplakin was elevated in the serum of paraneoplastic pemphigus patients before oral prednisolone treatment and decreased with a decline in disease activity after treatment (Ishii et al., 2012). The role of envoplakin in GCs treatment requires further investigation. IL-7 has been shown to induce GCs resistance *in vitro* in a subset of samples from patients with acute lymphoblastic leukemia (Olivas-Aguirre et al., 2021). In our study, the IL-7 receptor IL-7R was downregulated after GCs treatment in the treatment-effective group, suggesting that it plays a potential role in GCs resistance. Furthermore, guanine nucleotide-binding protein (G-protein) was downregulated in the treatment-effective group. G-proteins are a group of modulators or transducers that transmit signals from cell-surface receptors to generate physiological responses (Alvarez-Curto et al., 2016). Previous research revealed that the expression of specific G-protein subunits was under the coordinated control of GCs in the nervous system of rats, which demonstrated that G proteins are physiological targets of GCs *in vivo* (Saito et al., 1989). It has also been reported that the G protein βγ complex interacted with the GR and suppressed its transcriptional activity by associating with the transcriptional complex on GR-responsive promoters (Kino et al., 2005). Our study also suggested that G-protein is associated with GCs resistance, but the specific mechanism needs further clarification.

## Conclusion

In conclusion, we investigated the urine proteomic profiles in active non-segmental vitiligo patients with different GCs treatment responses pre and post treatment in this study and identified biomarkers for GCs treatment prediction and monitoring. The identified DEPs were found to be related to GCs signaling pathways and vitiligo pathogenic mechanisms. Before systemic steroid treatment, 242 DEPs were found between the treatment-effective and -resistant groups, and we established RBP-1 and TOR1AIP-1 as predictive biomarkers for GCs efficacy. Using samples collected at the follow-up visit, 341 proteins were found to be differentially expressed between these two groups. Our results identified RBP-1, TOR1AIP-1, and PDIA-4 as potential markers for treatment evaluation. This is the first attempt at applying urine proteomics to vitiligo, and our findings provide novel insights into steroid treatment efficacy prediction and steroid-resistance mechanisms.

## Data Availability

The datasets presented in this study can be found in online repositories. The mass spectrometry proteomics data have been deposited to the ProteomeXchange Consortium (http://proteomecentral.proteomexchange.org) via the iProX partner repository with the dataset identifier PXD031595. These data could be accessed by the following links: http://proteomecentral.proteomexchange.org/cgi/GetDataset?ID=PXD031595 https://www.iprox.cn/page/project.html?id=IPX0003738000.
