# Supplementary material for: Urinary Proteomics Analysis of Active Vitiligo Patients: Biomarkers for Steroid Treatment Efficacy Prediction and Monitoring
Source: Front Mol Biosci. 2022 Feb 17;9:761562. doi: 10.3389/fmolb.2022.761562 (PMC8891126; doi:10.3389/fmolb.2022.761562)

**Supplemental Figure 1.** Assessment of QC samples. The QC samples showed a stable condition with high correlation (R²=0.95).


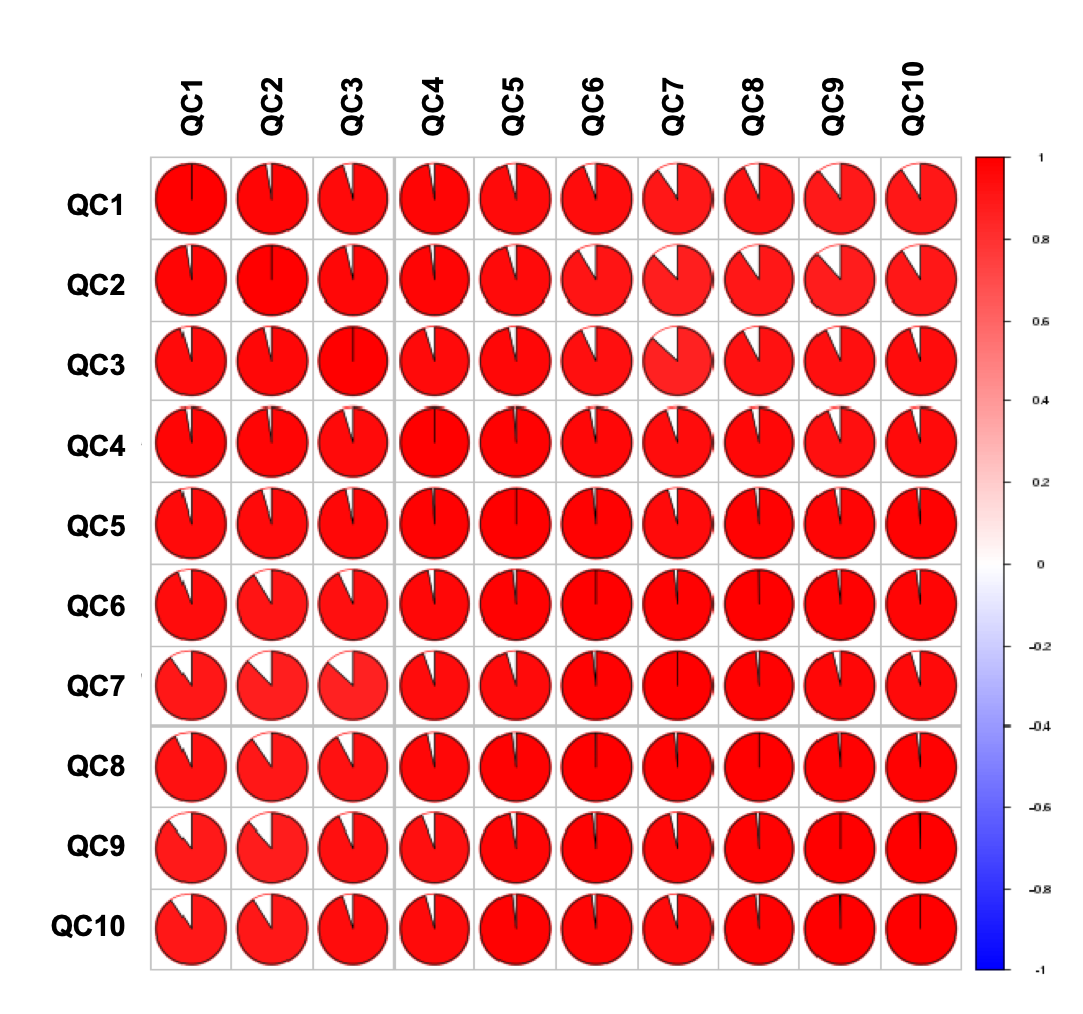


**Supplemental Figure 2.** PCA analysis of urine proteomics between treatment-resistant and treatment-effective active vitiligo patients before GCs **(A)**. 50 permutation validation plot for the OPLS-DA model **(B)**. (SIMCA 14.0 software, Umetrics, Sweden)


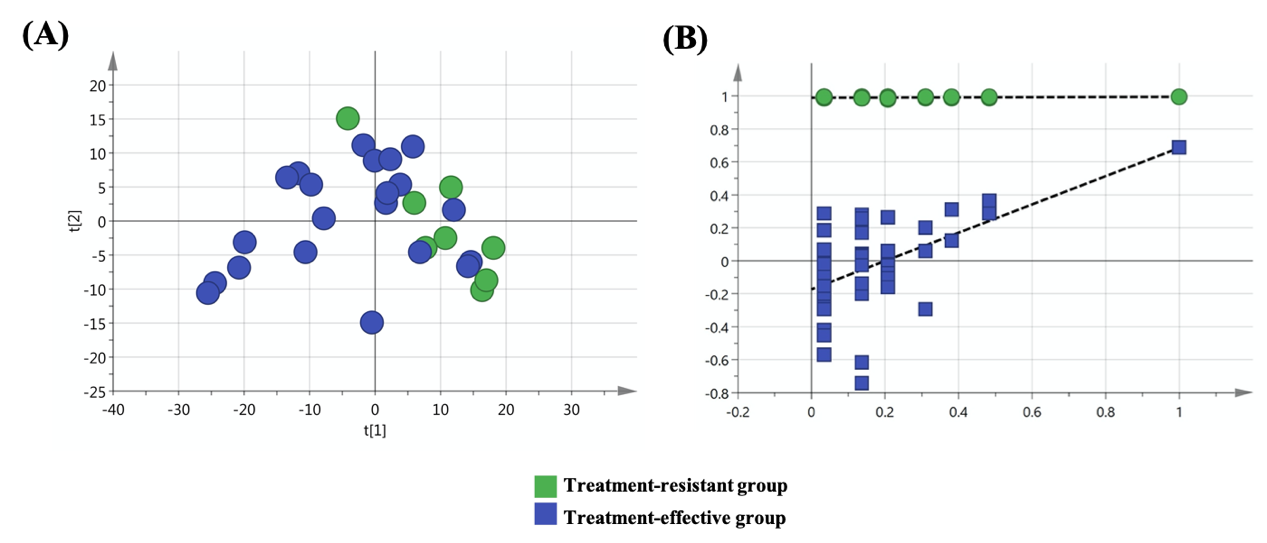


**Supplemental Figure 3.** PCA analysis of urine proteomics between treatment-resistant and treatment-effective active vitiligo patients after GCs **(A)**. 50 permutation validation plot for the OPLS-DA model **(B).** (SIMCA 14.0 software, Umetrics, Sweden)


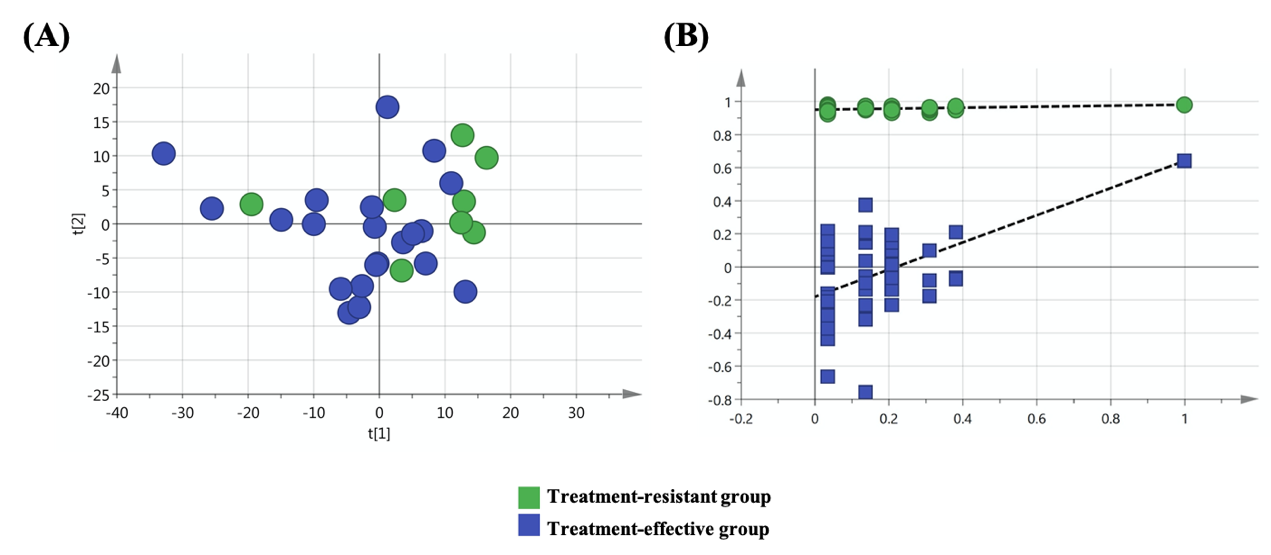


**Supplemental Figure 4.** The comparison of DEPs before **(A)** and after **(B)** treatment measured by ELISA and DIA analysis. The asterisks indicate the level of significance. *, P<0.05; **, P<0.01; ***, P<0.001; ns, no statistical difference. RBP-1, retinol binding protein-1; TOR1AIP-1, torsin 1A interacting protein-1; PDIA-4, protein disulfide-isomerase A-4; MAT2A, S-adenosylmethionine synthase isoform type-2; EVPL, evoplakin.

**
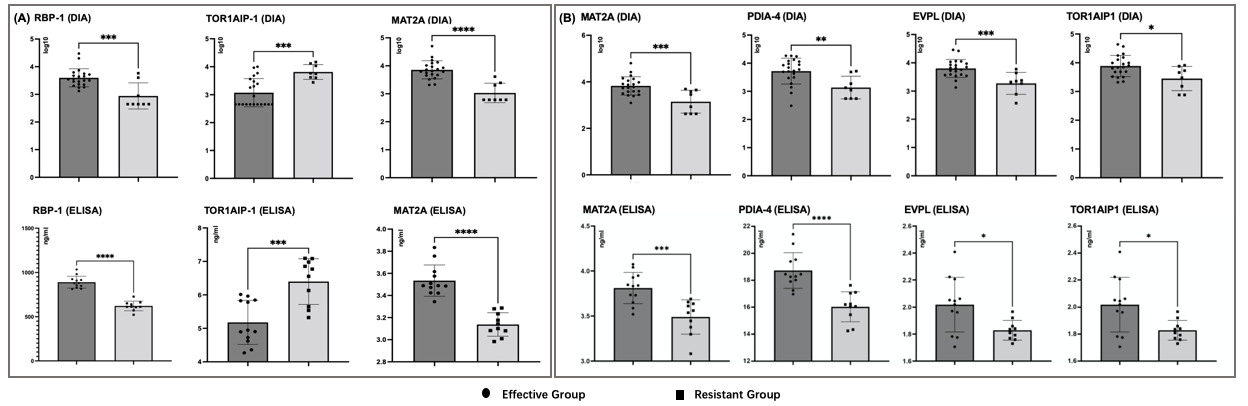
**

**Supplemental Figure 5.** The urinary level of differential proteins before **(A)** and after **(B)** treatment in different groups measured by enzyme-linked immunosorbent assay. The asterisks indicate the level of significance. *, P<0.05; **, P<0.01; ***, P<0.001; ns, no statistical difference. PDIA-4, protein disulfide-isomerase A-4; MAT2A, S-adenosylmethionine synthase isoform type-2; EVPL, evoplakin.


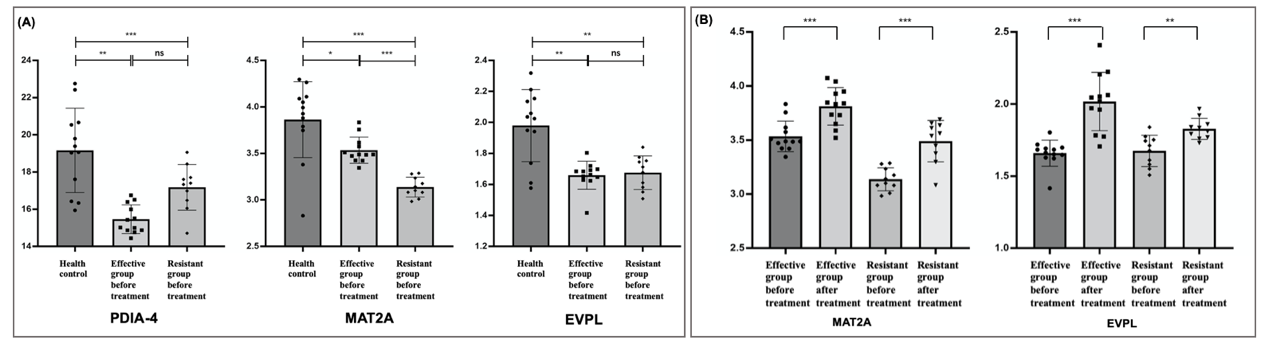

Supplement: Supplementary file 6 [file DataSheet1.docx]
